# Supplementary material for: Imidazolium Ionic Liquids as Compatibilizer Agents for Microcrystalline Cellulose/Epoxy Composites
Source: Polymers (Basel). 2023 Jan 9;15(2):333. doi: 10.3390/polym15020333 (PMC9865422; doi:10.3390/polym15020333)
Supplement: Supplementary file 1 [file polymers-15-00333-s001.zip › polymers-2139848-supplementary.pdf]

Imidazolium ionic liquids as compatibilizer agents for microcrystalline cellulose/epoxy composites

Eduardo Fischer Kerche[a,*], Vinícius Demétrio da Silva[b], Nicholas Alves Salles[a], Henri Stephan Schrekker[b], Sandro Campos Amico[a]

[a] PPGE3M, Federal University of Rio Grande do Sul – UFRGS, Av. Bento Gonçalves 9500, Porto Alegre, RS, 91501-970, Brazil.

[b] Laboratory of Technological Processes and Catalysis, Federal University of Rio Grande do Sul – UFRGS, Av. Bento Gonçalves 9500, Porto Alegre, RS, 91501-970, Brazil.

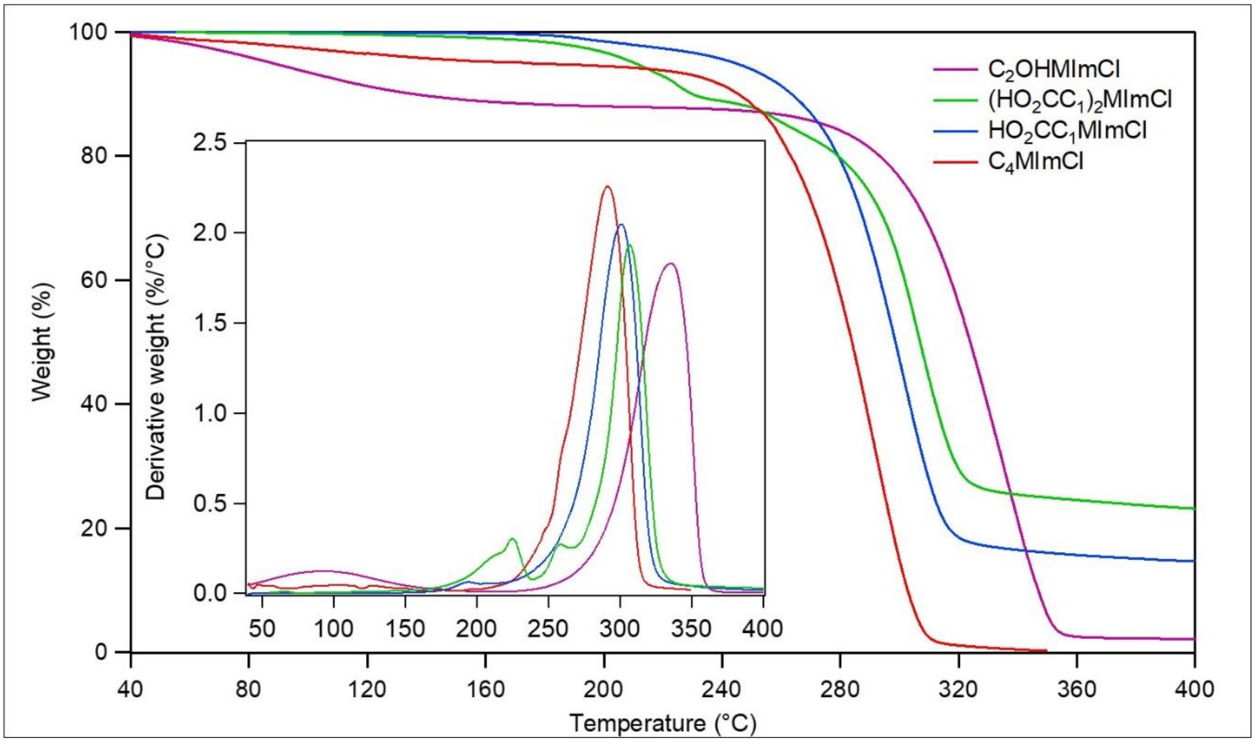

Figure S1: TG and DTG curves of **C_4MImCl** (red), **HO_2CC_1MImCl** (blue), **$(HO_2CC_1)MImCl$** (green) and **$C_2OHMImCl$** (purple), used as compatibilizers for the MCC/epoxy composites.

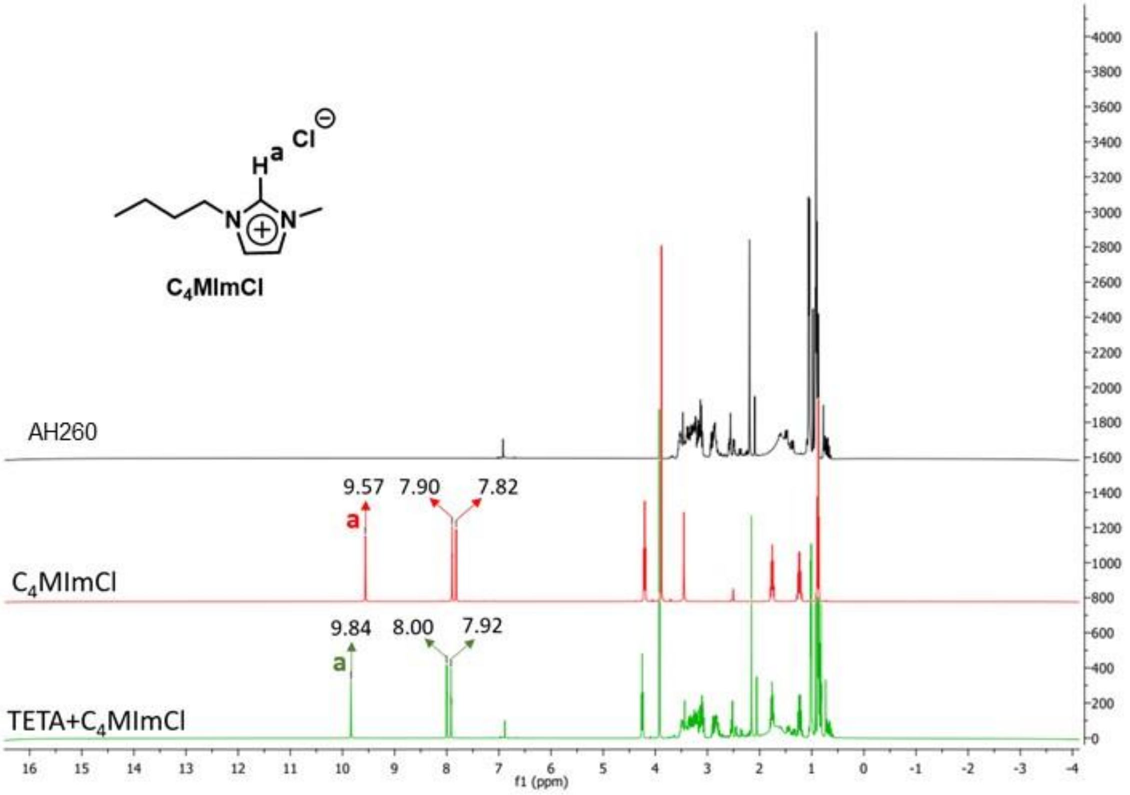

Figure S2: ^{1}H NMR spectra (400 MHz, DMSO-*d*6) of AH260 hardener (based on triethylenetetramine (TETA)) in black, **C_4MImCl** in red, and **C_4MImCl** + AH260 in green, where "a" represents the most acidic hydrogen of the imidazolium ring (C_2-H).

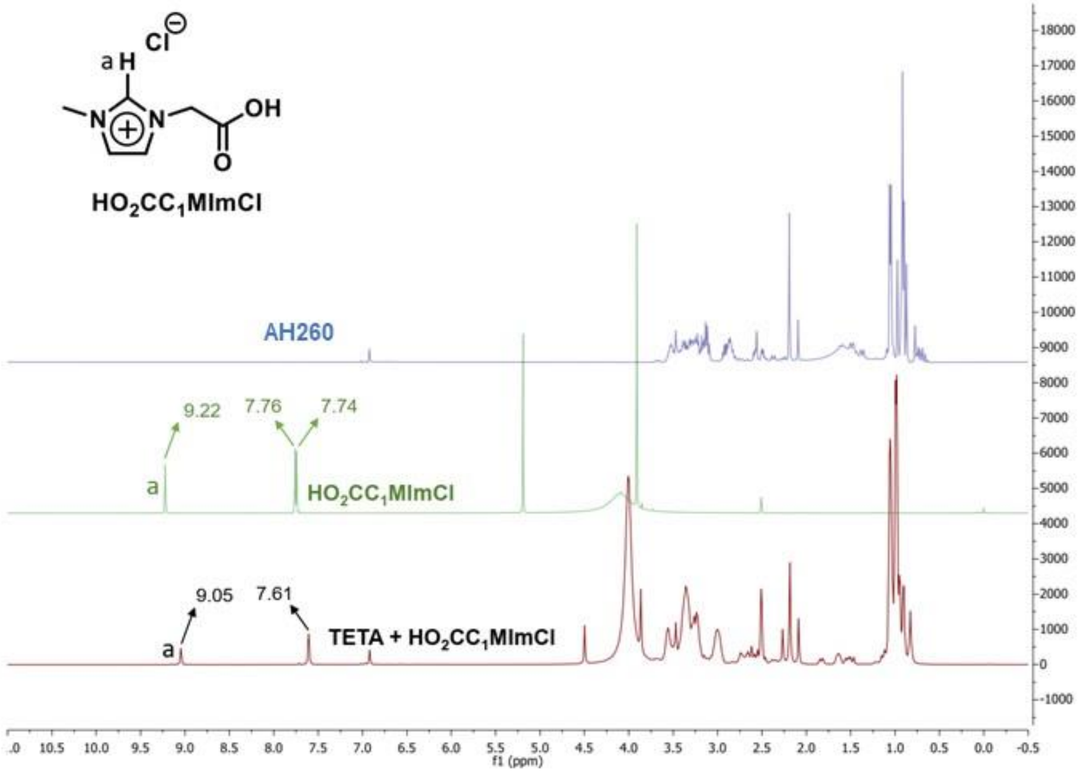

Figure S3: ^{1}H NMR spectra (400 MHz, DMSO-*d*6) of AH260 hardener (based on triethylenetetramine (TETA)) in blue, **HO_2CC_1MImCl** in green and **HO_2CC_1MImCl** + TETA in red, where "a" represents the most acidic hydrogen of the imidazolium ring (C_2-H).

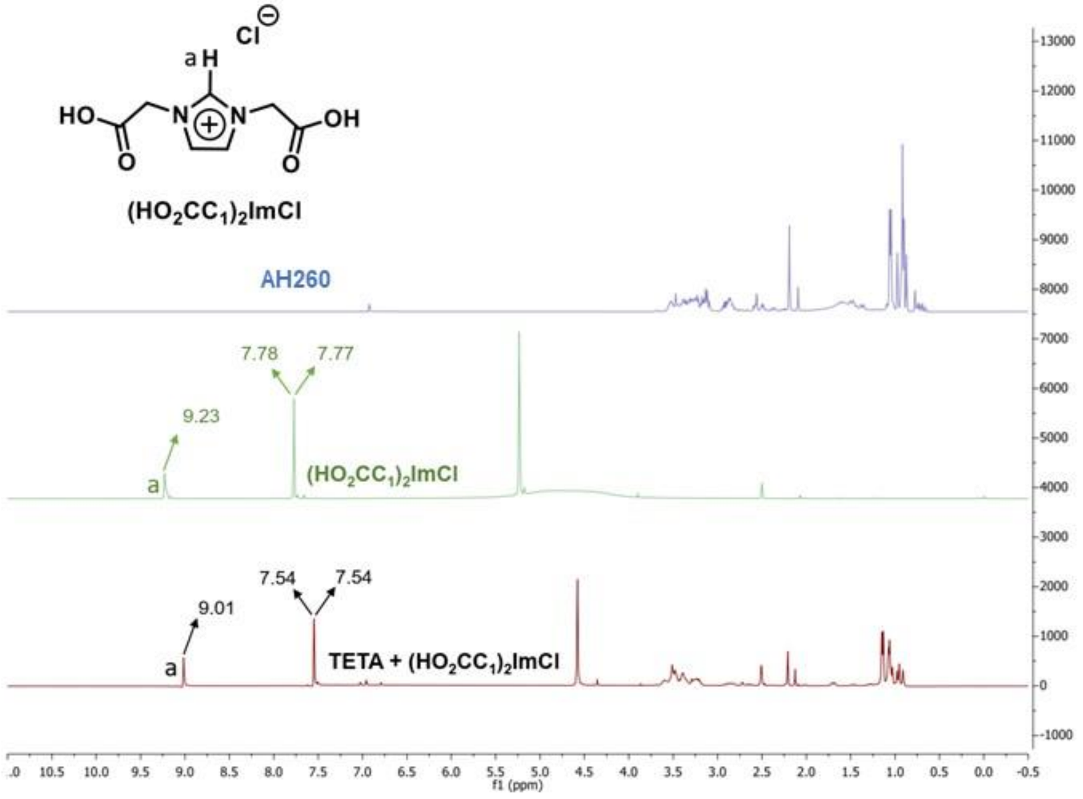

Figure S4: ^{1}H NMR spectra (400 MHz, DMSO-*d*6) of AH260 hardener (based on triethylenetetramine (TETA)) in blue, **(HO_2CC_1)$_2$MImCl** in green and **(HO_2CC_1)$_2$MImCl** + TETA in red, where "a" represents the most acidic hydrogen of the imidazolium ring (C_2-H).

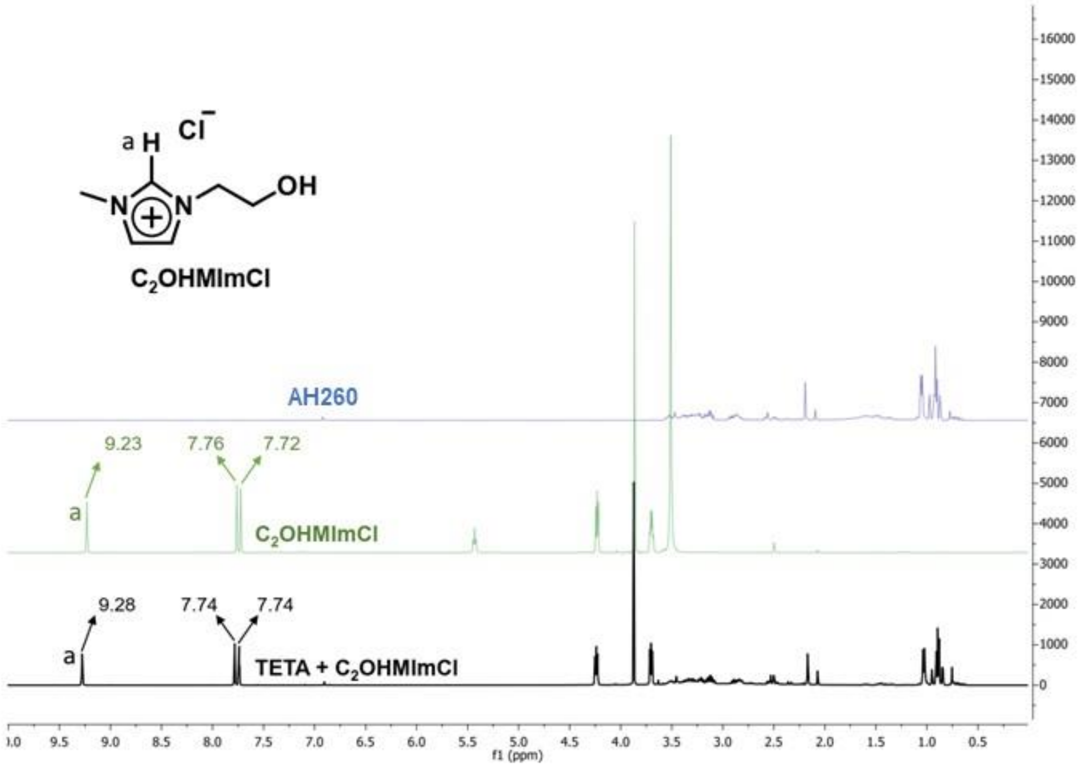

Figure S5: ^{1}H NMR spectra (400 MHz, DMSO-*d*6) of AH260 hardener (based on triethylenetetramine (TETA)) in blue, **C_2HOMImCl** in green and **C_2HOMImCl** + TETA in black, where "a" represents the most acidic hydrogen of the imidazolium ring (C_2-H).

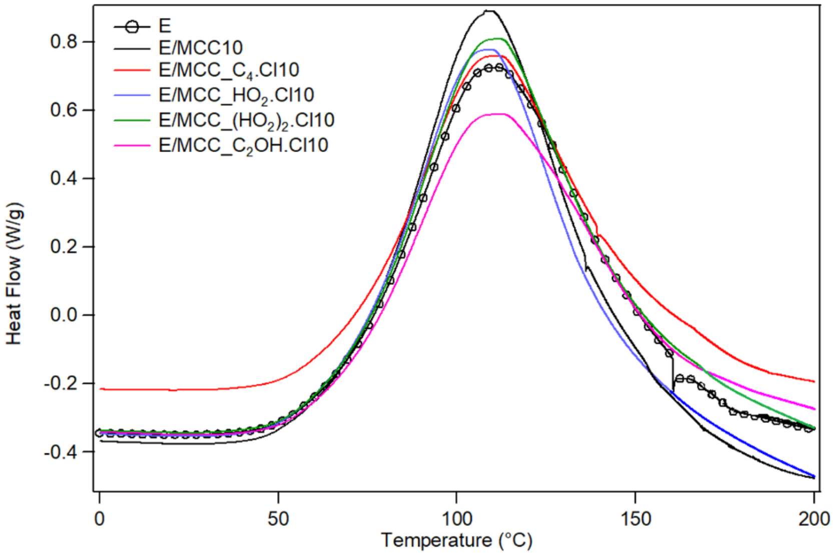

Figure S6: DSC curves for epoxy and the 10 phr MCC/epoxy composites.

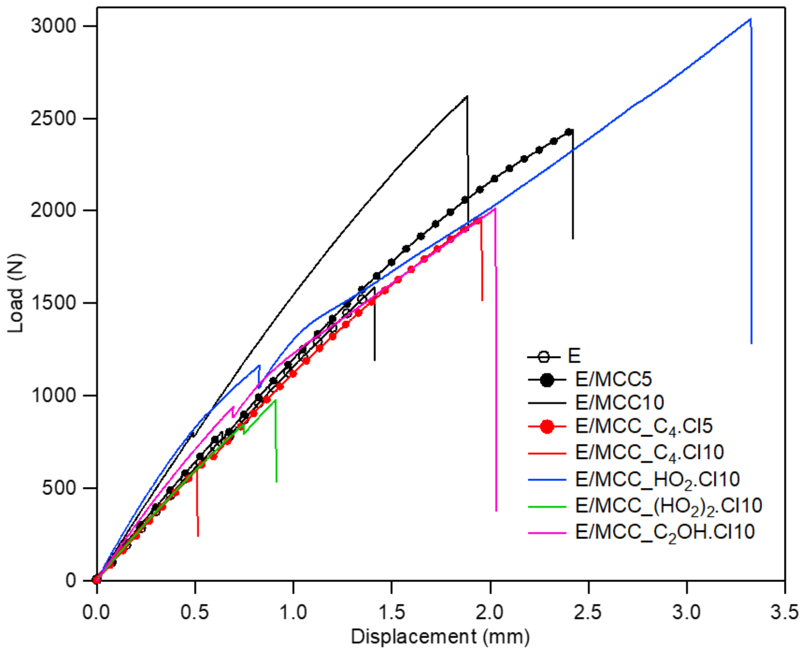

Figure S7: Median curves from tensile tests for epoxy and the MCC/epoxy composites.

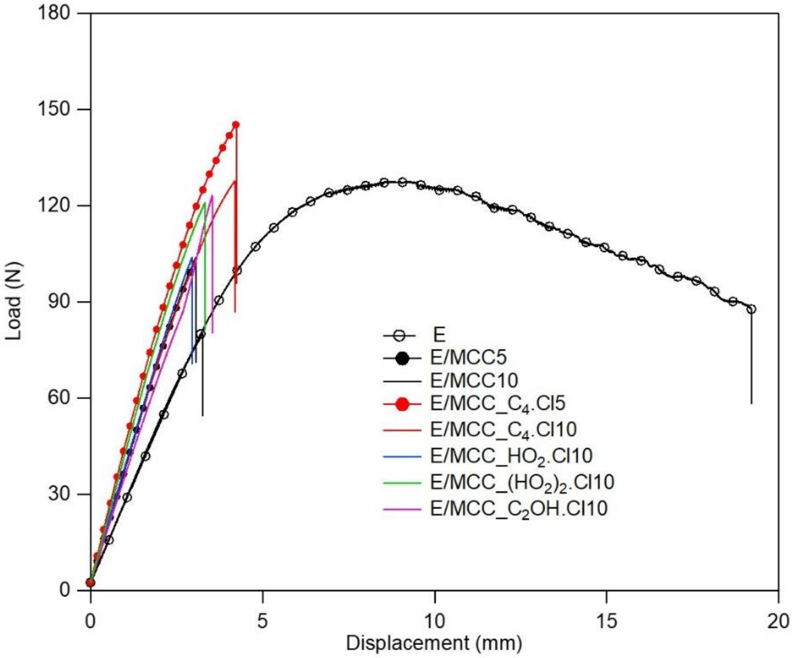

Figure S8: Median curves from 3-point bending tests for epoxy and the MCC/epoxy composites.
